# Neuroprotection of the hypoxic-ischemic mouse brain by human CD117^+^CD90^+^CD105^+^ amniotic fluid stem cells

**DOI:** 10.1038/s41598-018-20710-9

**Published:** 2018-02-05

**Authors:** Michelangelo Corcelli, Kate Hawkins, Filipa Vlahova, Avina Hunjan, Kate Dowding, Paolo De Coppi, Anna L. David, Donald Peebles, Pierre Gressens, Henrik Hagberg, Mariya Hristova, Pascale V. Guillot

**Affiliations:** 1 University College London, Institute for Women’s Health, Maternal and Fetal Medicine Department, London, UK; 2 University College London, Great Ormond Street Institute for Child Health, London, UK; 3 NIHR University College London Hospitals Biomedical Research Centre, Maple House, London, UK; 4 King’s College London, Department of Perinatal Imaging and Health, St. Thomas’ Hospital, London, UK; 5 Perinatal Center, Institutes of Clinical Sciences & Neuroscience and Physiology, Sahlgrenska Academy, Gothenburg University, Gothenburg, Sweden

## Abstract

Human amniotic fluid contains two morphologically-distinct sub-populations of stem cells with regenerative potential, spindle-shaped (SS-hAFSCs) and round-shaped human amniotic fluid stem cells (RS-hAFSCs). However, it is unclear whether morphological differences correlate with functionality, and this lack of knowledge limits their translational applications. Here, we show that SS-hAFSCs and RS-hAFSCs differ in their neuro-protective ability, demonstrating that a single contralateral injection of SS-hAFSCs into hypoxic-ischemic P7 mice conferred a 47% reduction in hippocampal tissue loss and 43–45% reduction in TUNEL-positive cells in the hippocampus and striatum 48 hours after the insult, decreased microglial activation and TGFβ1 levels, and prevented demyelination. On the other hand, RS-hAFSCs failed to show such neuro-protective effects. It is possible that SS-hAFSCs exert their neuroprotection via endoglin-dependent inhibition of TGFβ1 signaling in target cells. These findings identify a sub-population of CD117^+^CD90^+^CD105^+^ stem cells as a promising source for the neuro-protection of the developing brain.

## Introduction

Human fetal stem cells present a number of advantageous characteristics over their adult counterparts, such as faster growth kinetics, longer telomeres and higher differentiation potential^1^, thus presenting an intermediate phenotype between adult mesenchymal stem cells and embryonic stem cells^2^. Human amniotic fluid contains an alternative extraembryonic source of non-tumorigenic fetal stem cells that can be safely, ethically and readily isolated during ongoing pregnancy from consenting women undergoing mid-trimester clinical amniocentesis or from the fluid collected at time of delivery^3^. The regenerative potential of human amniotic fluid stem cells (hAFSCs)^4^ is further harnessed by their ability to give rise to multiple mesenchymal and non-mesenchymal lineages^5^ and their immunomodulatory properties^6^. However, the heterogeneity of the hAFSC population presents a hurdle for their therapeutic applications. Two distinct adherent cell types have previously been isolated by Roubelakis *et al*. based on their morphological properties, and described as spindle-shaped (SS) and round-shaped (RS) hAFSCs, with SS-hAFSCs representing about 6% of the total hAFSC population and RS-hAFSCs 94%^7^. Comparative analysis revealing the higher proliferation rate, differentiation potential and *in vitro* migration ability of SS-hAFSCs^7^. However, their *in vivo* regenerative potential has not been systematically compared and most pre-clinical experiments have been performed using heterogeneous populations of hAFSCs.

To determine whether the morphological and phenotypical differences observed *in vitro* between SS-hAFSCs and RS-hAFSCs correlate with differential functionality, we compared the neuro-repair potential of the cells using the Vannucci mouse model of neonatal hypoxic ischaemic encephalopathy (HIE)^8^, whereby the left common carotid artery is permanently occluded and the mice exposed to a 60 min hypoxic challenge in 8% oxygen. HIE is a major healthcare burden, being the fourth leading cause of childhood mortality and resulting in one million neuro-disabled children each year^9,10^. Whilst moderate hypothermia is now standard care, it is only partially effective and cannot be applied to pre-term babies^11^. With currently no effective cure addressing the underlying loss of cerebral tissue, there is an urgent need to develop a simple, safe and effective treatment to directly modulate pathophysiological processes and protect the developing brain of HIE-affected babies. Stem cell therapy has the potential to lessen brain injury either by replacing lost cells, promoting the differentiation of host progenitors, and/or modulating the host immune system^12,13^.

Here we show that, contrary to the results obtained with RS-hAFSCs, a single contralateral injection of SS-hAFSCs into the hypoxia-ischemia (HI) mouse brain contributed to lessening the size of the brain lesion, reduced the number of TUNEL^+^ cells, decreased microglial activation, prevented demyelination and reduced TGFβ1 levels. We are the first to demonstrate that SS-hAFSCs, which can easily be isolated from heterogeneous populations of hAFSCs based on their cell surface co-expression of CD105 and CD90, have potential for the protection of the developing brain.

## Results

### Differential cell surface molecule expression in SS-hAFSCs and RS-hAFSCs

**Figure 1:**
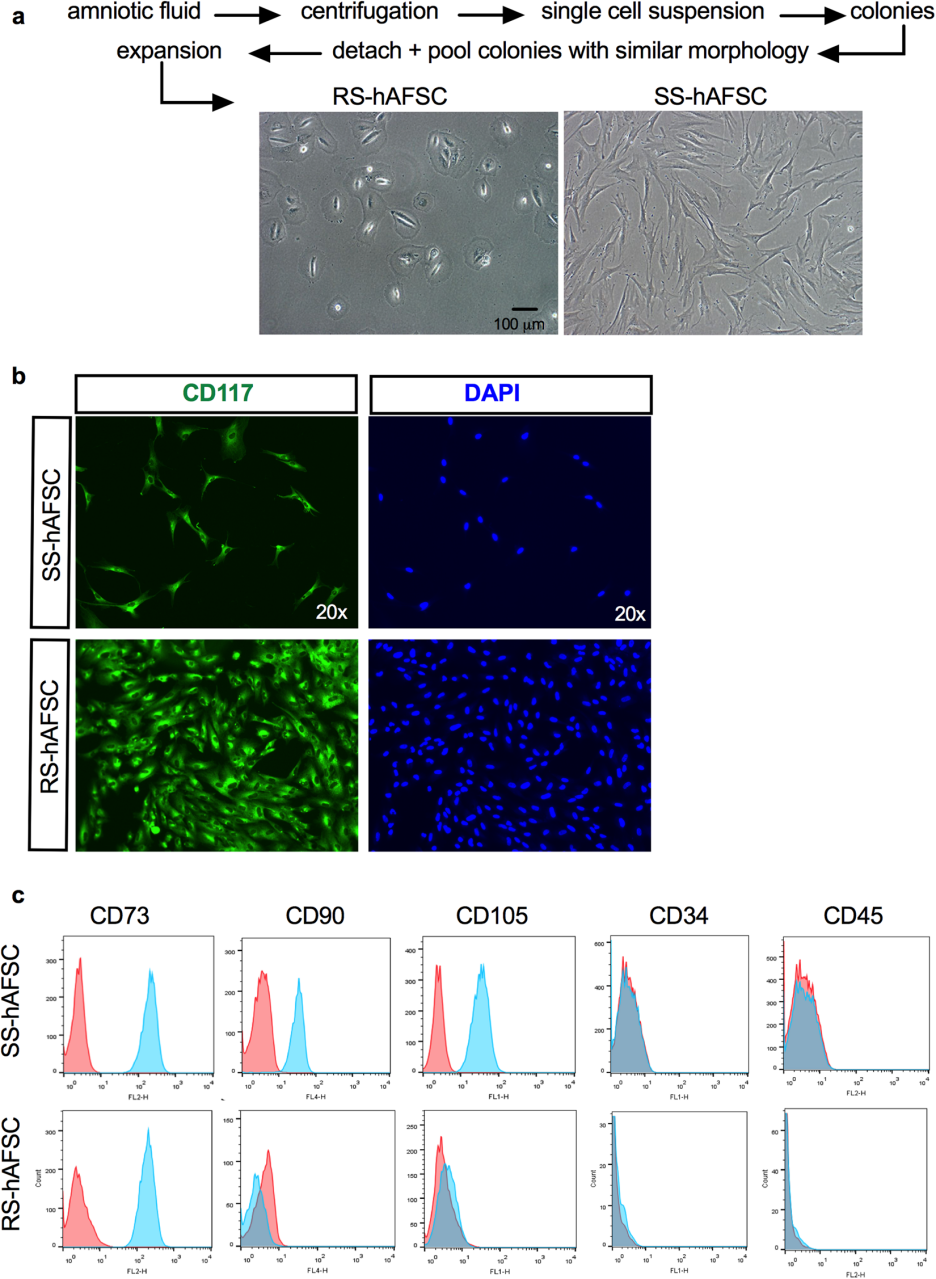
SS-hAFSCs and RS-hAFSCs differ for the expression of CD105 and CD90. (**a**) Isolation and representative inverted light microscopy images of human mid-trimester round-shaped (RS-hAFSCs) and spindle-shaped (SS-hAFSCs) amniotic fluid stem cells. (**b**) Example confocal immunostaining images demonstrating cytoplasmic CD117 staining (in green) in SS-hAFSCs and RS-hAFSCs counterstained with nuclear DAPI (blue). (**c**) Flow cytometry showing expression of CD73 and absence of expression of CD34 and CD45 in SS-hAFSCs and RS-hAFSCs, with only SS-hAFSCs expressing the cell surface markers CD90 and CD105. The red tracing shows the isotype control and the blue tracing shows the primary antibody.

The human mid-trimester amniotic fluid contains a heterogeneous population of plastic-adherent cells presenting either a polymorphic round-shaped appearance (RS-hAFSCs) or an elongated spindle-shaped cytoplasm (SS-hAFSCs) (Fig. 1a,b). The cells studied here were karyotypically normal, isolated from the same gestational age (2 samples16 weeks and 3 days of gestation and 2 samples 16 weeks of gestation) and pooled from four different donors undergoing prenatal diagnostic (healthy pregnancy) to reduce inter-individual variability. Both sub-populations were characterised by the positive expression of the stem cell marker CD117 in the cytoplasm (which is expressed on the cell surface of freshly isolated samples but gets internalised during *ex vivo* expansion) (Fig. 1b), the positive expression of the MSC marker CD73 and the negative expression of the hematopoietic markers CD34 and CD45 (Fig. 1c). However, contrary to SS-hAFSCs, RS-hAFSCs do not express the cell surface markers CD90 (Thy-1) and CD105 (endoglin) (results obtained from 7 samples of SS-hAFSCs and 6 samples of RS-hAFSCs), indicating that these surface markers can also be used for purification of heterogeneous cell adherent CD117^+^ (C-Kit^+^) human amniotic fluid stem cell populations.

### SS-hAFSCs, but not RS-hAFSCs, prevent lesion of the HI mouse hippocampus

**Figure 2:**
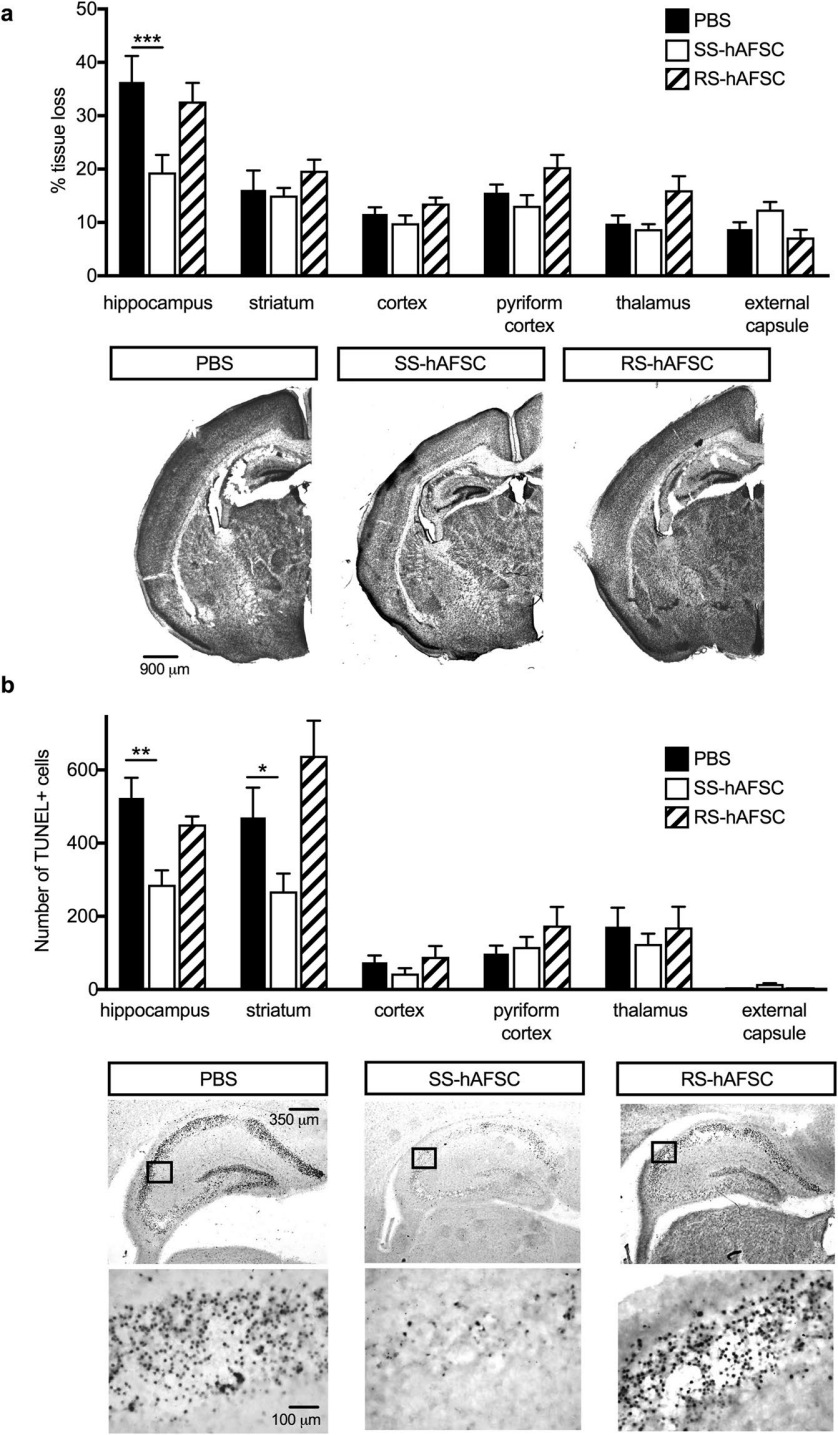
SS-hAFSCs decrease hippocampal lesion and number of dead cells in hippocampus and striatum. (**a**) Quantification of brain tissue volume loss in contralateral forebrain, assessed by Nissl staining (Cresyl-Violet), showing reduction of infarct size in the hippocampus of HI mice injected with SS-hAFSCs (mean ± SEM), ***P < 0.001, * < 0.05. Representative images of Nissl-stained brain sections of HI mice injected with either PBS only, SS-hAFSCs or RS-hAFSCs. **(b**) TUNEL + staining showing reduced number of dying cells in HI brains injected with SS-hAFSCs. (mean ± SEM), **P < 0.01, *P < 0.05. Representative images (low and high magnification) of TUNEL-stained brain sections of HI mice injected with either PBS only, SS-hAFSCs or RS-hAFSCs.

To determine whether the differences in morphology and immunophenotype of SS-hAFSCs and RS-hAFSCs translate into differential regenerative functionality, we injected a single dose of 1 × 10^5^ stem cells in 20 μl PBS in the contralateral ventricle of P7 HI mice immediately after the HI insult, induced by the permanent ligation of the left carotid followed by exposure to 8% oxygen to induce hypoxic conditions. We compared the histology of the brain of HI mice injected with stem cells with those injected with PBS only (without stem cells) at 48 hours following HI insult. Histological analysis of the HI brain injected with PBS only revealed brain tissue loss compared to the control side in all the cerebral regions assessed (hippocampus, striatum, cortex, pyriform cortex, thalamus and external capsule) with the hippocampus being the structure with the largest lesion size (Fig. 2a), (uninjured control group in Supplementary Figure 1a). Injection of SS-hAFSCs led to a significant (47%) reduction in infarct size in the hippocampus (19.3 ± 3.2, n = 16) compared to HI mice injected with PBS alone (36.4 ± 4.8, mean ± SEM, n = 20; P = 0.00008 two-way ANOVA followed by Bonferroni’s multiple comparison test), but no difference was observed between PBS-injected mice and mice injected with SS-hAFSCs in the striatum (16.1 ± 3.6, n = 20 vs. 15.1 ± 1.4; P = 0.99), cortex (11.6 ± 1.2, n = 20 vs. 9.8 ± 1.4, n = 16; P = 0.99), pyriform cortex (15.6 ± 1.5, n = 20 vs. 13.1 ± 1.9, n = 16; P = 0.99), thalamus (9.8 ± 1.5, n = 20 vs. 8.7 ± 0.9, n = 16; P = 0.77) and external capsule (8.8 ± 1.3, n = 20 vs. 12.4 ± 1.4, n = 16; P = 0.99). Notably, no difference was observed between PBS-injected mice and mice injected with RS-hAFSCs in any of the brain regions tested, i.e. the hippocampus (32.7 ± 3.4, n = 16; P = 0.98), striatum (19.7 ± 2.1, n = 16; P = 0.99), cortex (13.6 ± 1.2, n = 16; P = 0.99), pyriform cortex (20.4 ± 2.3, n = 16; P = 0.99), thalamus (10.1 ± 2.6, n = 16; P = 0.99) and external capsule (7.2 ± 1.4, n = 16; P = 0.99).

### Injection of SS-hAFSCs, but not RS-hAFSCs, decreases the number of TUNEL-positive cells in the hippocampus and striatum

Histological analysis of the HI brains injected with PBS only, showed that the HI insult resulted in the presence of TUNEL + staining of dying brain cells with fragmented DNA 48 hours later, with the hippocampus and striatum being the most affected regions (Fig. 2b), (uninjured control group in Supplementary Figure 1b). Compared to PBS-injected mice, the injection of SS-hAFSCs led to a 45% decrease of TUNEL + cells in the hippocampus (523.6 ± 55.1 mean ± SEM, n = 9 vs. 286.2 ± 39.7, n = 14; P = 0.007, two-way ANOVA followed by Bonferroni’s multiple comparison test) and to a 43% decrease in the striatum (470.3 ± 81.16, n = 9 vs. 268.2 ± 48.8, n = 14; P = 0.048), but not in the cortex (74.6 ± 18.3, n = 9 vs. 43.7 ± 14.5, n = 14; P = 0.99), pyriform cortex (98.11 ± 21.7, n = 8 vs.116.4 ± 27.2, n = 13; P = 0.99), thalamus (171.6 ± 52.2 vs.124.6 ± 27.9, n = 14; P = 0.99) or external capsule (2.6 ± 0.4, n = 12 vs. 15.1 ± 2.3, n = 14; P = 0.99). Notably, injection of RS-hAFSCs had no significant positive effect on the hippocampus (451.3 ± 21.4, n = 15; P = 0.98), striatum (638.8 ± 95.7, n = 14; P = 0.14), cortex (89.6 ± 29.5, n = 16; P = 0.99), pyriform cortex (174.9 ± 50.6, n = 15; P = 0.97), thalamus (171.6 ± 52.2, n = 6; P = 0.99) and external capsule (2.6 ± 0.4, n = 12; P = 0.99).

### Injection of SS-hAFSCs decreases microglial activation

**Figure 3:**
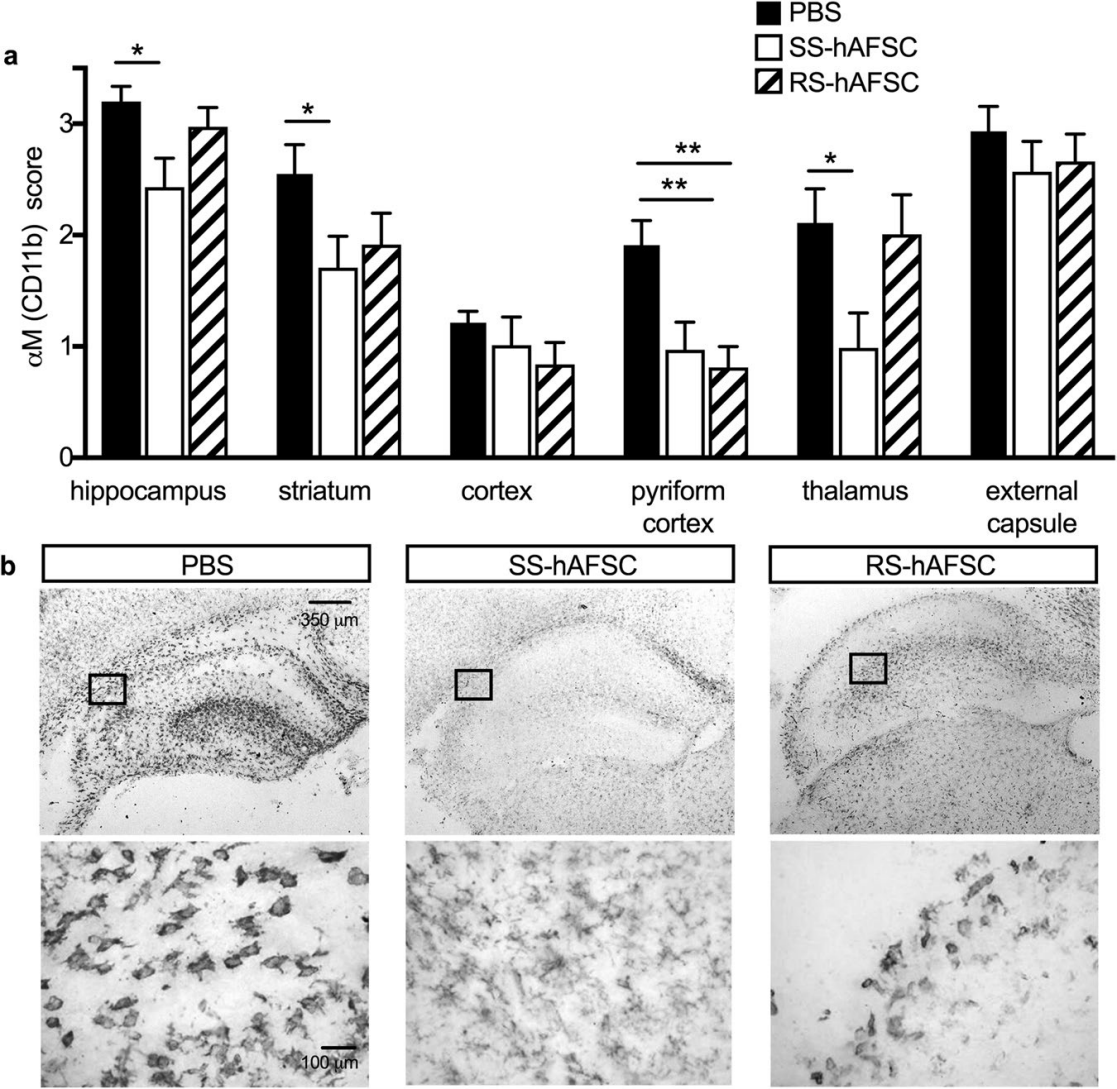
SS-hAFSCs reduce microglia activation in hippocampus, striatum and pyriform cortex. (**a**) Microglia activation, assessed by αM (CD11b) scoring in contralateral forebrain, showing reduction in the hippocampus, striatum and pyriform cortex of HI mice injected injected with SS-hAFSCs compared to brains injected with PBS alone without cells (mean ± SEM), **P < 0.01, *< 0.05. **(b**) Representative images (low and high magnification) of CD11b-stained brain sections of HI mice injected with either PBS only, SS-hAFSCs or RS-hAFSCs.

To analyse the mechanistic role of donor stem cells, we assessed microglia activation by the expression of CD11b (αM) (Fig. 3a) and cell morphology (Fig. 3b), (uninjured control group in Supplementary Figure 1c). HI insult led 48 hours later to strong microglia activation. We showed that, in comparison to PBS-injected brains, injection of SS-hAFSCs significantly decreased CD11b expression in the hippocampus (3.2 ± 0.1, n = 20 vs. 2.4 ± 0.2, n = 16; P = 0.01, two-way ANOVA followed by Bonferroni’s multiple comparison test), striatum (2.5 ± 0.2, n = 20 vs. 1.7 ± 0.2, n = 16; P = 0.03), pyriform cortex (1.9 ± 0.2, n = 22 vs. 1.0 ± 0.2, n = 16; P = 0.01) and thalamus (2.1 ± 0.3, n = 22 vs. 0.9 ± 0.3, n = 16; P = 0.01), whilst injection of RS-hAFSCs only decreased CD11b in the pyriform cortex (1.9 ± 0.2, n = 22 vs. 0.8 ± 0.2, n = 16; P = 0.01) (Fig. 3a). However, SS-hAFSCs injection had no effect on CD11b expression in the cortex (1.2 ± 0.1, n = 22 vs. 1.0 ± 0.2, n = 16; P = 0.06) and external capsule (2.9 ± 0.2 vs. 2.5 ± 0.3, n = 16; P = 0.98), whilst RS-hAFSC had no effect in the hippocampus (3.2 ± 0.1 vs. 3.0 ± 0.2, n = 15; P = 0.99), striatum (2.5 ± −0.2 vs. 1.9 ± 0.3, n = 14; P = 0.61), cortex (1.2 ± 0.1 vs. 0.8 ± 0.2, n = 16; P = 0.97), thalamus (2.1 ± 0.3, n = 22 vs. 2.0 ± 0.3, n = 15; P = 0.99) and external capsule (2.9 ± 0.2 vs. 2.7 ± 0.2, n = 16; P = 0.99) (Fig. 3a). We observed more amoeboid or round morphology in the microglia of brains injected with either PBS alone or with RS-hAFSCs, compared to the ramified morphology^14^ in the brains injected with SS-hAFSCs (Fig. 3b).

### Both SS-hAFSCs and RS-hAFSCs decrease reactive astrogliosis

**Figure 4:**
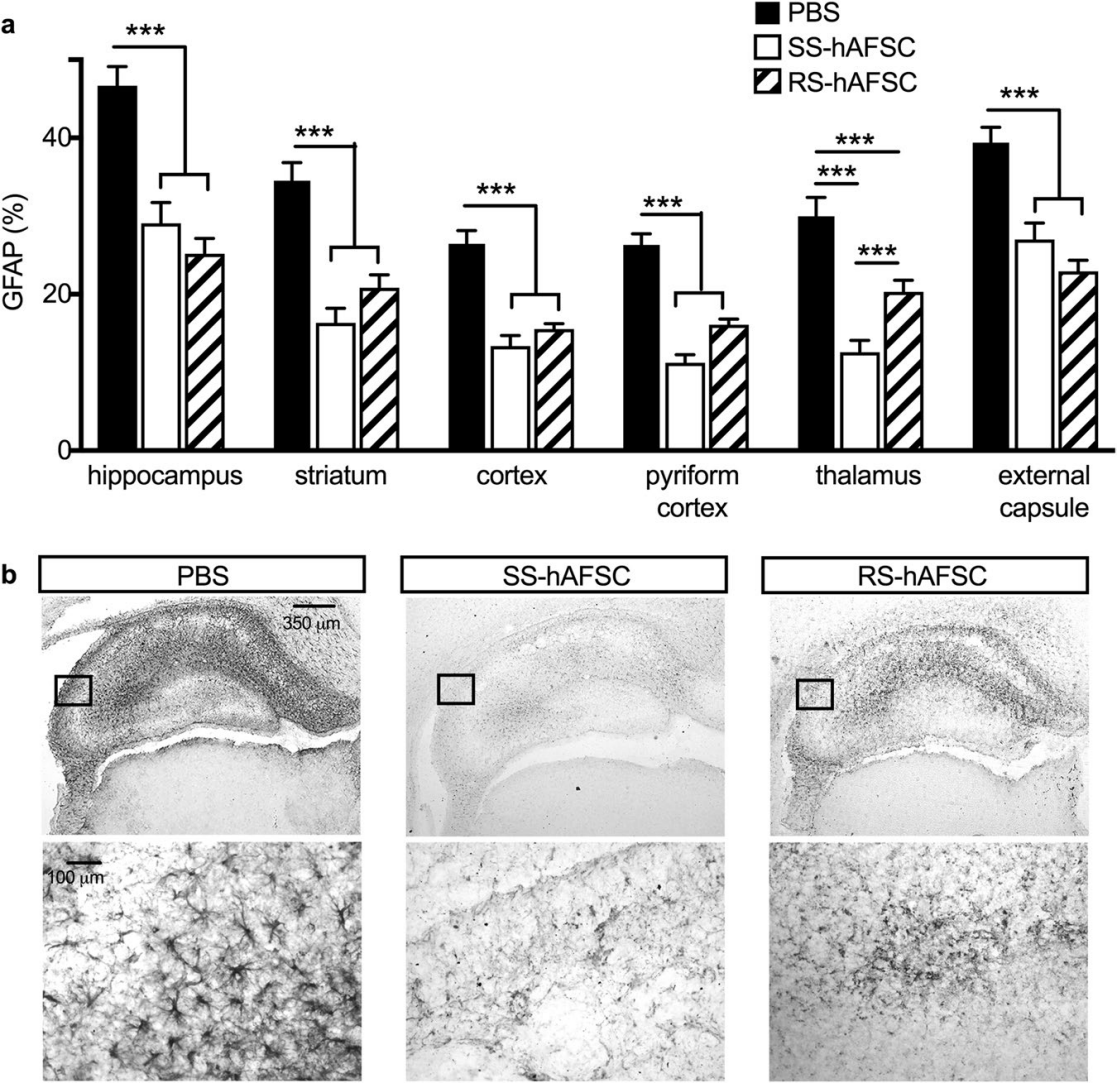
Both SS-hAFSCs and RS-hAFSCs reduce reactive astrogliosis. (**a**) Reactive astrogliosis, assessed by GFAP staining in contralateral forebrain, showing reduction in all the brain regions of HI mice injected with SS-hAFSCs or RS-hAFSCs, compared to PBS-injected brains (mean ± SEM), ***P < 0.0001. **(b**) Representative images (low and high magnification) of GFAP-stained brain sections of HI mice injected with either PBS only, SS-hAFSCs or RS-hAFSCs.

Next, to investigate the impact of donor stem cells on reactive astrogliosis 48 h after injection, we determined the percentage of cells expressing GFAP (Fig. 4), (uninjured control group in Supplementary Figure 1d). Interestingly, both stem cell subsets significantly reduced GFAP immunoreactivity in all regions of the brain analysed. Compared to PBS-injected mice, those injected with either SS-hAFSCs or RS-hAFSCs decreased astroglial activation in the hippocampus (46.7 ± 2.4 mean ± SEM, n = 22 vs. 29.1 ± 2.6, n = 16 vs. 25.2 ± 2.6, n = 16; F(2.51) = 24.46, P = 0.00008, two-way ANOVA), striatum (34.5 ± 2.3, n = 22 vs. 16.3 ± 1.8, n = 16 vs. 20.8 ± 1.6, n = 16; F(2,51) = 22.58, P < 0.0001), cortex (26.4 ± 1.7, n = 22 vs. 13.4 ± 1.3, n = 16 vs. 15.4 ± 0.7, n = 16; F(2,51) = 25.92, P < 0.0001), pyrocortex (26.3 ± 1.4, n = 22 vs. 11.2 ± 1.0, n = 16 vs. 16.1 ± 0.7, n = 16; F(2,51) = 44.4, P < 0.0001), thalamus (29.9 ± 2.4, n = 22 vs. 12.5 ± 1.5, n = 16 vs. 20.3 ± 1.5, n = 16; F(2,51) = 19.1, P < 0.0001) and external capsule (39.4 ± 1.9, n = 22 vs. 27.0 ± 2.1, n = 16 vs. 22.9 ± 1.4, n = 16; F(2,51) = 21.4, P < 0.0001). Compared to the intense GFP staining in PBS-injected brains, those injected with either SS-hAFSCs or RS-hAFSCs showed reduced staining intensity, with those injected with SS-hAFSCs showing the strongest decrease (Fig. 4b).

### Injection of SS-hAFSCs prevents demyelination in the striatum and external capsule

**Figure 5:**
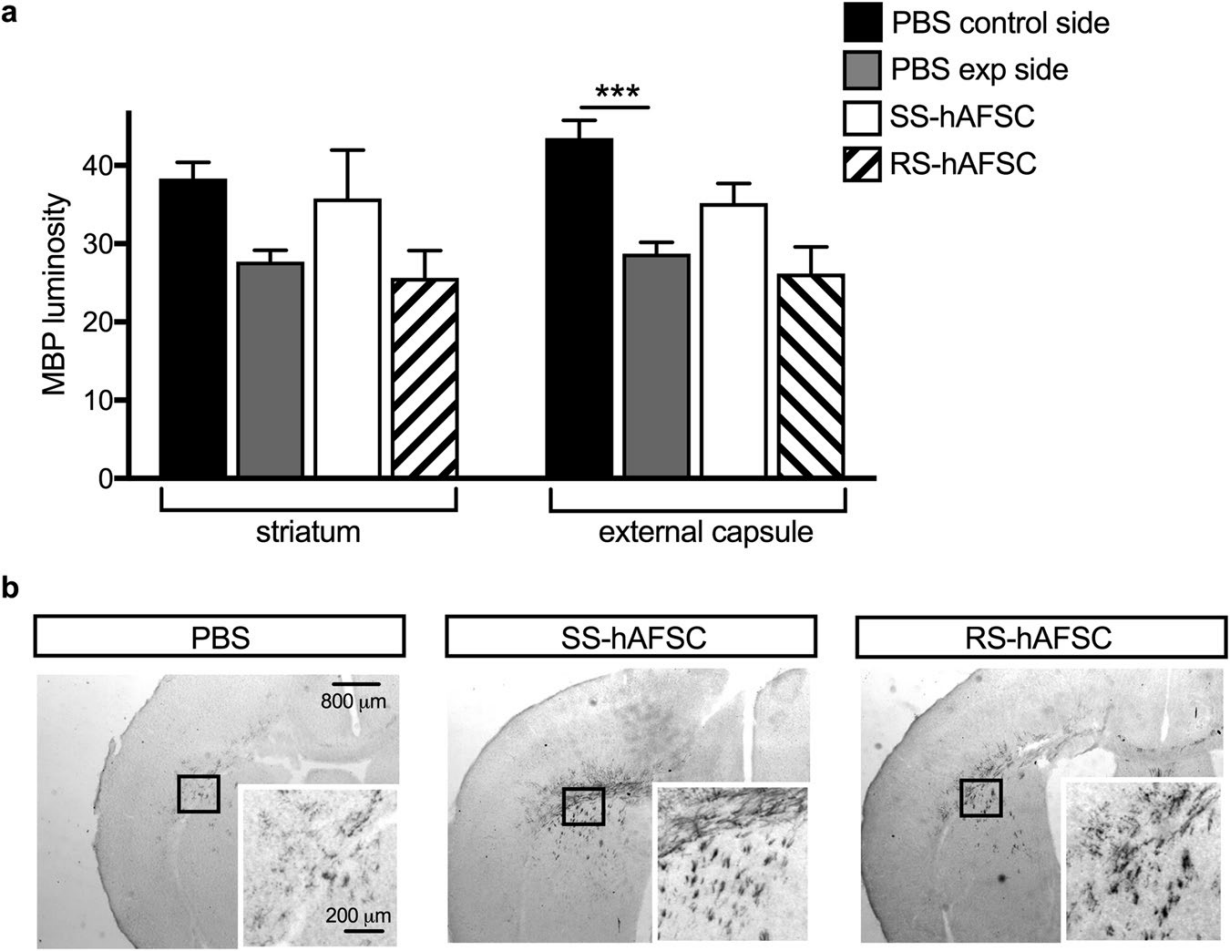
SS-hAFSC injection prevents demyelination in the striatum and external capsule. (**a**) Myelin Binding Protein luminosity in contralateral forebrain, showing increase in the striatum and external capsule of HI mice injected with SS-hAFSCs, compared to PBS-injected brains (mean ± SEM), ***P < 0.0001. **(b**) Representative images (low and high magnification) of MBP-stained brain sections of HI mice injected with either PBS only, SS-hAFSCs or RS-hAFSCs.

Neuronal myelination was assessed by measuring the intensity of myelin basic protein (MBP) immunostaining in the striatum and external capsule. Results revealed that in PBS-injected brains, the HI insult significantly decreased MBP luminosity in the external capsule (43.5 ± 2.3, n = 10 vs. 28.7 ± 1.5, n = 18, P = 0.0007, two-way ANOVA followed by Bonferroni’s multiple comparison test), but was not significant in the striatum (38.3 ± 2.1 mean ± SEM, n = 11 vs. 27.7 ± 1.5, n = 18; P = 0.21) (Fig. 5a). However, injection of SS-hAFSCs tended to prevent demyelination in both regions (35.8 ± 6.1, n = 14; P = 0.18 in the striatum and 35.2 ± 2.5, n = 14; P = 0.21 in the external capsule). Notably, the injection of RS-hAFSCs did not show any positive effect on myelination in either brain regions (25.6 ± 3,4, n = 15; P = 0.99 in the striatum and 26.1 ± 3.4, n = 15; P = 0.99 in the external capsule) (Fig. 5a). Immunohistochemistry for MBP confirmed the trend towards prevention of demyelination in the HI brains injected with SS-hAFSCs (Fig. 5b).

### Injection of SS-hAFSCs led to a 65% decrease in TGFβ1^+^ cells

**Figure 6:**
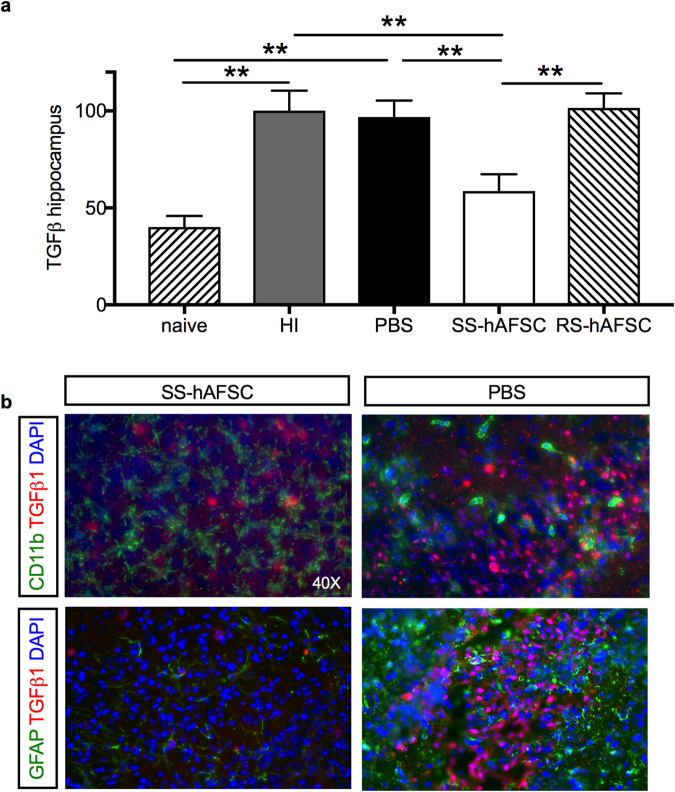
Hippocampal TGFβ1 levels are reduced in mice transplanted with SS-hAFSCs. (**a**) Number of TGFβ1^+^ cells (average of three random fields with 200 × magnification) in the hippocampus of naïve mice, HI non-transplanted mice, and in the hippocampus of HI mice transplanted with either PBS alone without stem cells, SS-hAFSCs and RS-hAFSCs. (mean ± SEM), **P < 0.001. (**b**) Co-immunostaining for GFAP (green staining), TGFβ1 (red staining) and DAPI (blue) and CD11b (green staining), TGFβ1 (red staining) and DAPI (blue) in the hippocampus of HI mice transplanted with SS-hAFSCs and PBS only.

The number of hippocampal TGFβ1 + cells was significantly increased in the brains of mice that received an HI insult and were not injected (HI) and those who received an HI insult but were injected with PBS alone (PBS), compared to those that did not receive any HI insult (naïve) (100.1 ± 10.3, n = 9 vs. 96.9 ± 8.5, n = 17 vs. 40.2 ± 5.7, n = 6; P = 0.003, one-way ANOVA followed by Bonferroni’s multiple comparison test). Injection of SS-hAFSCs, but not RS-hAFSCs, led to a 65% decrease in TGFβ1^+^ cells (58.6 ± 8.6, n = 16; P = 0.005 for SS-hAFSCs and 101.6 ± 7.4, n = 15, P > 0.99 for RS-hAFSCs) (Fig. 6a). Co-immunostaining for TGFβ1 and either CD11b (microglia) or GFAP (astroglia) on sections of PBS-injected brains confirmed reduced levels of TGFβ1, CD11b and GFAP levels in SS-hAFSC- transplanted mice, as shown in Fig. 6b.

### Neuroprotection of the HI mouse brain by SS-AFSCs is likely to be via endoglin-dependent inhibition of TGFβ1

We injected 100,000 cells in 2 μl PBS in the contralateral side of the brain and assessed the presence of donor cells in the ipsilateral side 48 hours later using an anti-human Nuclear Antigen antibody, which reacts with human but not mouse tissue. We found that donor SS-hAFSCs or RS-hAFSCs were absent in the ipsilateral side of the brain, suggesting that the beneficial effects observed by donor cells are not mediated via direct differentiation but through the release of soluble factors.

**Figure 7:**
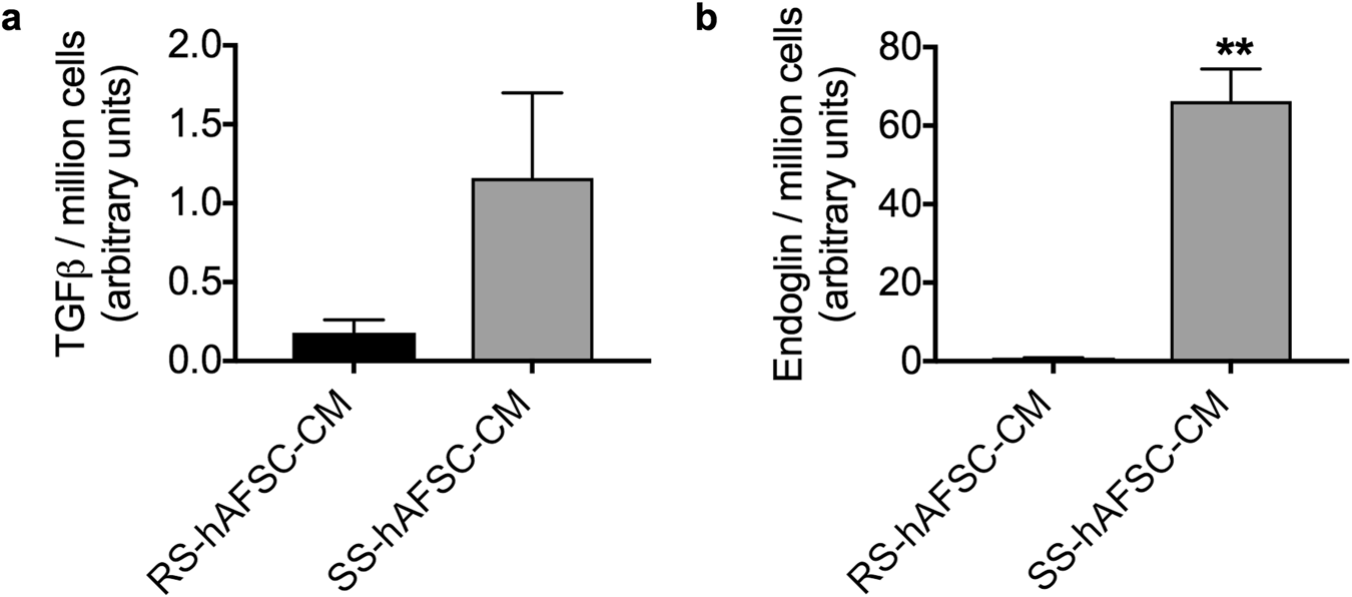
Potential mechanism of action of SS-hAFSC in the hypoxic-ischemic mouse brain. **(a)** TGFβ1 ELISA demonstrates that both SS-hAFSCs and RS-hAFSCs secrete TGFβ1. (**b**) Endoglin ELISA demonstrates increased levels of secretion in SS-hAFSC compared to RS-hAFSC. **p < 0.01.

Transforming growth factor (TGF) β1 secreted by MSCs has been recently shown to modulate the functional properties of microglia, suggesting that TGFβ1 levels may be used as a biological marker to predict the efficacy of stem cells to counteract neuro-inflammation^15^. However, the concentration of TGFβ1 released by SS-hAFSCs was not significantly higher than that released by RS-hAFSCs (0.18 ± 0.08, mean ± SD, n = 4 for RS-hAFSCs vs. 1.16 ± 0.54, n = 4 for SS-hAFSCs, unpaired t-test, P = 0.12) (Fig. 7a). However, endoglin (CD105), a glycoprotein that binds TGFβ1 with high affinity and inhibits TGFβ1-smad3 signalling^16^, was released by SS-hAFSCs but not by RS-hAFSCs (66.2 ± 8.2, n = 4 mean ± SD for SS-hAFSCs vs. 0.54 ± 0.3, n = 4 for RS-hAFSCs, t-test, P = 0.0002) (Fig. 7b). Together, these results indicate that whilst both SS-hAFSCs and RS-hAFSC secrete TGFβ, only SS-hAFSCs secrete endoglin, which may inhibit TGFβ1-smad3 signalling in target cells.

## Discussion

Human amniotic fluid, which can be retrieved during caesarean section or at birth, is a valuable source of fetal stem cells that can be used without ethical concerns for allogeneic and autologous applications in regenerative medicine. These cells present a higher ability to differentiate and exhibit higher growth kinetics than adult stem cells, whilst showing no sign of tumorigenicity^3^. Despite having been characterised by their plastic-adherence and expression of the stem cell surface marker CD117, human amniotic fluid stem cells (hAFSCs) are heterogeneous with regards to their morphology. We, and others, have in particular distinguished between cells presenting a spindle-shaped (SS-hAFSCs) and round-shaped (RS-hAFSCs) morphology, which show differences with regards to their proteomic profile, growth characteristics and differentiation potential^7,17^. In the present study, we isolated and pooled clonal populations of SS-hAFSCs and RS-hAFSCs and showed that both cell types express CD73 but, unlike RS-hAFSCs, SS-hAFSCs uniquely co-express CD90 (Thy-1) and CD105 (endoglin). Thy-1 has several cellular functions, being involved in cell-cell interactions, regulating apoptotic signalling, modulating proliferation, survival and cytokine/growth factor response, and promoting T cell activation^18^. Endoglin is part of the TGFβ receptor complex, thus modulating the response to the binding of TGFβ, activin A and BMPs. It plays an important role in angiogenesis^19^. However, there is no evidence that phenotypical differences translate into *in vivo* functional differences. Here, we show that a single injection of SS-hAFSCs into the HI mouse brain immediately after the HI insult resulted 48 hours later in a significant reduction of brain lesion size and TUNEL-positive cells. Notably, injection of RS-hAFSCs failed to show such neuro-protective effects. Donor cells were absent from the site of injury 48 hours after intracranial injection, suggesting they mediate their neuro-repair effects through paracrine/endocrine mechanisms. In this study, we pooled together several donor samples, to reduce inter-individual differences. Further analysis will be required to *develop in vitro* screening platforms to compare the therapeutic efficacy of individual samples. The HI mouse model used in this study, which is a modification of the Vannucci model of unilateral ligation/occlusion of the common carotid artery at postnatal day 7 followed by a period of hypoxia, presents some phenotypic similarities to periventricular white matter damage in humans. These similarities include prevention of demyelination, ventricular enlargement, loss of neurons, damage to axons and dendrites, oligodendrocyte cell death and alterations of oligodendrocyte development, which are the hallmarks of brain injury in preterm/term infants^20^. We are the first to report the heterogeneity of the hAFSC population in relation to its neuro-regenerative properties, and to identify a sub-population of CD117+ spindle-shaped cells that can be easily isolated and purified based on their spindle-shape morphology and co-expression of CD90 and CD105.

In the specific disease model used here, we demonstrated that understanding the mechanistic properties of the cells is important to further improve stem cell-based therapy. Specifically, our data show that both cell types decreased reactive astrogliosis, indicating that the neuro-protective effects of SS-hAFSCs are not attributable to a reduction in astroglial activation and indeed astrogliosis failed to influence on the extent of HI brain injury in neonatal mice^20^. However, SS-hAFSCs, but not RS-hAFSCs, lowered the levels of astroglial TGFβ1, decreased microglia activation and prevented demyelination, suggesting that donor SS-hAFSCs modulated the host immune system by decreasing resident TGFβ1 levels, thereby lowering the microglial response to neuronal death and restoring the oligodendrocyte/astrocyte differentiation balance. Not surprisingly, further analysis of the phenotypical differences between SS-hAFSCs and RS-hAFSCs showed that only SS-hAFSCs release endoglin. These results indicate that inhibition of the TGFβ1 signaling pathway in target cells may be mediated by the release of endoglin.

In conclusion, this study provides the first evidence for the neuro-protective effects of a subset of spindle-shaped CD73^+^ CD90^+^ CD105^+^ fetal stem cells that can be easily and ethically isolated from mid-trimester amniotic fluid and will provide the basis for the development of innovative stem-cell based therapeutics for the protection of the developing brain.

## Methods

### Ethics statement

The healthy donors who provided the amniotic fluid in this study provided written informed consent in accordance with the Declaration of Helsinski. The ethical approval given by the Research Ethics Committees of Hammersmith & Queen Charlotte’s Hospitals (2001/6234) in compliance with UK national guidelines (Review of the Guidance on the Research Use of Fetuses and Fetal Material (1989), also known as the Polkinghorne Guidelines. London: Her Majesty’s Stationery Office, 1989: Cm762) for the collection of fetal tissue for research.

All experimental protocols were approved by the UK Home Office guidelines (PPL 70/7173 and 70/8784), and The Institutional Licensing Committee of University College London and we followed The ARRIVE guidelines. All methods were carried out in accordance with relevant guidelines and regulations.

### Cell culture

Human mid-trimester amniotic fluid was collected at mid-gestation from normal pregnancy and immediately spun before the cell pellet was resuspended into a single cell suspension in StemMACS MSC expansion media under xeno- and serum-free conditions at low density (10^2^ cells/cm^2^) onto a plastic culture dish and let to expand until clones of >50 cells formed. The clones had a homogeneous morphology, with some clones presenting a round-shaped cytoplasm, whilst others presented a spindle-shaped one. 5–10 clones presenting similar morphology were mechanically detached, pooled, spun, resuspended into single cells and plated (10^4^ cells/cm^2^) on plastic culture dishes without feeders in Dulbecco’s modified Eagle’s medium (DMEM-HG) (Invitrogen) supplemented with 10% fetal bovine serum (Biosera), 2 mM L-glutamine, 50 IU/ml penicillin and 50 mg/ml streptomycin (Gibco-BRL) (D10), at 37 °C in a 5% CO_2_ incubator.

### Enzyme-linked immunosorbent assays (ELISAs)

Cells were plated at 2 × 10^4^ cells/cm^2^ in four wells of a 6-well plate. 72 hours after seeding, 8 ml conditioned medium was removed and concentrated using an Amicon ultra-4 15 ml 3 K NMWL filter by centrifugation at 4000 g for 40 minutes. 300 µl of D10 was then added to the concentrated medium and 100 µl of this was added per well of the ELISA plate. The human TGFβ1and endoglin ELISA kit (Abcam) were performed according to the manufacturer’s instructions.

### Flow cytometry

Cells were detached as described previously and washed in flow buffer (PBS + 1% BSA). Cells were then centrifuged at 5000 g for 2 minutes before 1 × 10^5^ cells were resuspended in the appropriate primary antibody (anti-CD105, anti-CD90, anti-CD73, [All Miltenyi Biotec]) at its optimal dilution (1:10) in flow buffer and incubated for 1 hour at 4 °C. For unconjugated antibodies, cells were then washed and resuspended in a 1:10 dilution of FITC-conjugated donkey anti-mouse (Jackson ImmunoResearch labs) for 30 minutes at 4 °C. Cells were then analysed using a Becton Dickinson FACScalibur flow cytometer (BD bioscience) using Cell Quest Pro and FlowJo software.

### Immunofluorescence

Cryosections were post-fixed in 4% PFA in Phosphate-Buffered Saline (PBS 1 × pH 7.6; 5 min, RT), rinsed in PBS, blocked (1 h RT) with PBS supplemented with 1% BSA and 5% Normal Goat Serum (NGS) andincubated (overnight, 4 °C) with the primary antibody TGFβ1 (1:200, Abcam ab92486), GFAP (1:500, Dako), CD11b (1:500, Serotec) in PBS/BSA, washed (3 times) in PBS/BSA, incubated (1 h RT) with secondary antibodies in PBS/BSA, washed and rinsed in PBSPBS and mounted inVectaShield containing DAPI (Vector Labs) and visualized immediately., Images were collected using a LeicaDM 6000 fluorescence microscope (40× PLAN APO objective) and transferred to Adobe Photoshop (Adobe Systems). Secondary antibodies for single and double immunofluorescence were: donkey anti-mouse IgG FITC (1:100 dilution; multiple-labelling grade; Jackson ImmunoResearch Laboratories) and goat anti-rabbit Alexa-Fluor 594, Thermo Fisher).

### Hypoxic-ischemic insult

The animals used were from the C57/BL6 strain (Charles River, Kent, UK). The surgical procedures to induce HI insult were performed as previously described^21^. Briefly, seven-day-old mice were anaesthetised with isofluorane (5% induction followed by 1.5% maintenance) before undergoing permanent left common carotid artery occlusion with 8/0 polypropylene suture. The mice were left to recover at 36 °C and returned to the dam for 2 hours before being placed in a hypoxia chamber and exposed to humidified 8% oxygen /92% nitrogen for 1 hour (severe insult). Mice were randomized to receive either 100,000 cells (SS-hAFSCs or RS-hAFSCs) in 2 μl PBS or with PBS only (with no cells) via freehand intracranial injection through the skull in the brain hemisphere contralateral to the arterial occlusion. The mice were then returned to the dam and sacrificed 48 hours later by intraperitoneal injection of pentobarbitone and perfused with 30 ml 4% paraformaldehyde in PBS (intracardiac injection). Mice from at least five different litters were used in each experimental group, and mice from each litter were randomly assigned to all experimental groups, taking gender into account in a way that both genders were equally distributed among groups.

### Tissue sample preparation

Brains were removed straight after perfusion, post-fixed in 4% paraformaldehyde in PBS (1 h, 4 °C) and cryopreserved in phosphate-buffered 30% sucrose solution for 24 hours, as previously described^21^. The brains were then frozen on dry ice, cut on a cryostat into sequential 40 μm sections and stored at −80 °C until processed for analysis by an experimenter blinded to the group analysed.

### Immunohistochemistry and histological analysis

For each brain, five cryosections (400 μm apart) were rehydrated in distilled water and stained using immunohistochemistry. The sections were incubated overnight with primary antibodies, i.e. CD11b (1:5000, Serotec), GFAP (1:6000, Dako), MBP (1:1000), washed with PBS, incubated for 2 hours with biotinylated goat anti-rabbit or anti-rat secondary antibody (1:100, Vector, Peterborough, UK), washed with PBS, and incubated with Avidin-Biotinylated horseradish peroxidase Complex (Vector) before being visualized with diaminobenzidin/H_2_O_2_ (Fisher Scientific, Loughborough, UK). Five further 400 μm-interspaced sections from each brain were used for Terminal transferase mediated d-UTP nick end labelling (TUNEL) (Roche, Burgess Hill, UK), following the manufacturer’s instructions. Five further 400 μm-interspaced sections from each brain were used for staining with cresyl violet (Nissl).

Detection of donor cells in the ipsilateral side of the brain was performed 48 hours after injection in the contralateral side using anti-human Nuclear Antigen antibody (Abcam).

### Scoring and measurement

The brain infarct volume was measured on Nissl-stained sections, as previously reported^20^. Briefly, sections scans were imported into the Optimas 6.5 image analysis software (Bothell, WA, USA) and areas of intact staining were outlined and measured bilaterally in cortex, hippocampus, striatum, pyriform cortex, thalamus and external capsule. The percentage of tissue loss was the calculated by converting the measured areas into square millimetres and converting the volume by multiplying by 400 μm. The sum of these volumes was used to calculate the percentage of intact brain tissue as injured/intact volume x 100.

For CD11b (αM integrin) scoring, semi-quantitative scores were allocated to each brain region (cortex, hippocampus, striatum, pyriform cortex, thalamus and external capsule) by an observer blinded to the treatment of the groups, as previous described^21,23^, to describe microglial appearance i.e. 0 (no activation), 1 (focal microglial activation), 2 (mild phagocytic activation affecting <50% of the region), 3 (phagocytic activation affecting >50% of the region) and 4 (total phagocytic activation).

For TUNEL immunoreactivity, the number of positive cells was counted bilaterally in three different optical fields at 20× magnification in cortex, hippocampus, striatum, pyriform cortex, thalamus and external capsule, and the counts were averaged per animal and per group.

The intensity of GFAP staining was quantified using optical luminosity values. Images for both ipsilateral and contralateral sides of the brain were captured with a Sony AVT-Horn 3CCD colour video camera (24 bit RGB, 760 × 570 pixel resolution) and three different optical fields in the cortex, hippocampus, striatum, pyriform cortex, thalamus and external capsule, as well as the surrounding glass at x20 magnification. We used Optimas 6.5 software to obtain the mean and standard deviation (SD) for optical luminosity values (OVL). SD was subtracted from the values obtained for the surrounding glass, as previously described^22^.

### Statistical analysis

Statistical analysis was performed using GraphPad 7.0b. Two-way ANOVAs, followed by Bonferroni’s multiple comparison post-hoc test, were used to test if there was an interaction between variables and obtain p values. One-way ANOVA were used to determine if there was any statistical difference between the means of more than two independent groups. Unpaired T-test was used to compare the means of two independent groups. P < 0.05 was considered significant.

## Electronic supplementary material

Supplementary Figure 1

## References

[1] Guillot PV, Gotherstrom C, Chan J, Kurata H, Fisk NM (2007). Human first-trimester fetal MSC express pluripotency markers and grow faster and have longer telomeres than adult MSC. Stem Cells.

[2] Moschidou D (2013). Molecular signature of human amniotic fluid stem cells during fetal development. Curr Stem Cell Res Ther.

[3] De Coppi P (2007). Isolation of amniotic stem cell lines with potential for therapy. Nat Biotechnol.

[4] Abdulrazzak H, De Coppi P, Guillot PV (2013). Therapeutic potential of amniotic fluid stem cells. Curr Stem Cell Res Ther.

[5] Resca E (2015). Enrichment in c-Kit improved differentiation potential of amniotic membrane progenitor/stem cells. Placenta.

[6] Di Trapani M (2015). Immune regulatory properties of CD117(pos) amniotic fluid stem cells vary according to gestational age. Stem Cells Dev.

[7] Roubelakis MG (2011). *In vitro* and *in vivo* properties of distinct populations of amniotic fluid mesenchymal progenitor cells. J Cell Mol Med.

[8] Vannucci RC, Vannucci SJ (1997). A model of perinatal hypoxic-ischemic brain damage. Ann N Y Acad Sci.

[9] Roubelakis MG, Tsaknakis G, Pappa KI, Anagnou NP, Watt SM (2013). Spindle shaped human mesenchymal stem/stromal cells from amniotic fluid promote neovascularization. PLoS One.

[10] Lee ACC (2013). Intrapartum-related neonatal encephalopathy incidence and impairment at regional and global levels for 2010 with trends from 1990. Pediatr Res.

[11] Robertson NJ (2012). Which neuroprotective agents are ready for bench to bedside translation in the newborn infant?. J Pediatr.

[12] Fleiss B (2014). Stem cell therapy for neonatal brain injury. Clin Perinatol.

[13] Bennet L (2012). Cell therapy for neonatal hypoxia-ischemia and cerebral palsy. Ann Neurol.

[14] Thored P (2009). Long-term accumulation of microglia with proneurogenic phenotype concomitant with persistent neurogenesis in adult subventricular zone after stroke. Glia.

[15] Noh, M. Y. *et al*. Mesenchymal stem cells modulate the functional properties of microglia via TGFb secretion. *Stem Cells Transl Med* 1538–1549 (2016). 10.5966/sctm.2015-0217 PMC5070497 27400795

[16] Guo B (2004). CD105 inhibits transforming growth factor-beta-Smad3 signalling. Anticancer Res.

[17] Roubelakis MG (2007). Molecular and proteomic characterization of human mesenchymal stem cells derived from amniotic fluid: comparison to bone marrow mesenchymal stem cells. Stem Cells Dev.

[18] Rege TA, Hagood JS (2006). Thy-1, a versatile modulator of signaling affecting cellular adhesion, proliferation, survival, and cytokine/growth factor responses. Biochim Biophys Acta.

[19] Bautch VL (2017). Endoglin moves and shapes endothelial cells. Nat Cell Biol.

[20] Jarlestedt K (2010). Attenuation of reactive gliosis does not affect infarct volume in neonatal hypoxic-ischemic brain injury in mice. PLoS One.

[21] Kendall GS, Robertson NJ, Iwata O, Peebles D, Raivich G (2006). N-methyl-isobutyl-amiloride ameliorates brain injury when commenced before hypoxia ischemia in neonatal mice. Pediatr Res.

[22] Moller JC (1996). Regulation of thrombospondin in the regenerating mouse facial motor nucleus. Glia.

[23] Hristova M (2016). Inhibition of Signal Transducer and Activator of Transcription 3 (STAT3) reduces neonatal hypoxic-ischaemic brain damage. J Neurochem.

