# Supplementary material for: Neuroprotection of the hypoxic-ischemic mouse brain by human CD117+CD90+CD105+ amniotic fluid stem cells
Source: Sci Rep. 2018 Feb 5;8:2425. doi: 10.1038/s41598-018-20710-9 (PMC5799160; doi:10.1038/s41598-018-20710-9)
Supplement: Supplementary file 1 — Supplementary Figure 1 [file 41598_2018_20710_MOESM1_ESM.pdf]

Neuroprotection of the hypoxic-ischemic mouse brain by human $CD117^{+}CD90^{+}CD105^{+}$ amniotic fluid stem cells.

Michelangelo Corcelli, Kate Hawkins, Filipa Vlahova, Avina Hunjan, Kate Dowding, Paolo De Coppi, Anna L David, Donald Peebles, Pierre Gressens, Henrik Hagberg, Mariya Hristova and Pascale V Guillot

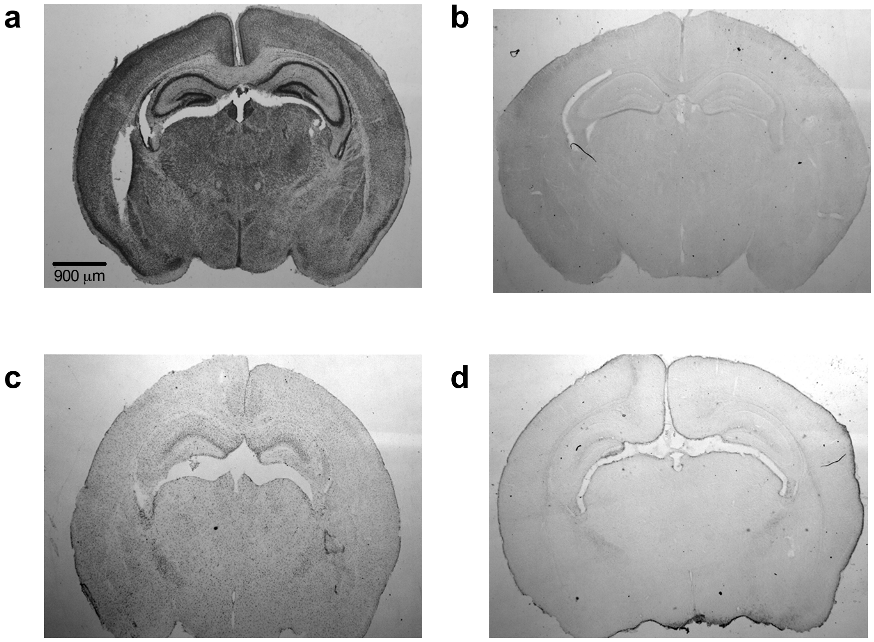

Supplementary Figure 1 | Uninjured control goup. (a) Cresyl violet (NISSL), (**b)** TUNEL+ cell death, **(c)** CD11b, **(d)** GFAP.
